# Key elements and theoretical logic of application of PIC/S standards in improving drug GMP inspections in China: an analysis based on grounded theory

**DOI:** 10.3389/fphar.2025.1559004

**Published:** 2025-08-11

**Authors:** Di Hu, Ping Liao, Yuwen Chen

**Affiliations:** ^1^ School of Business Administration, Shenyang Pharmaceutical University, Shanghai, China; ^2^ Shanghai Food and Drug Packaging Materials Control Center, Shanghai, China; ^3^ Shanghai Center for Drug Evaluation and Inspection, Shanghai, China

**Keywords:** PIC/S, China drug GMP inspection, grounded theory, inspectors, inspection procedures, inspection mutual trust

## Abstract

**Objective:**

Currently, China regulatory authorities are committed to improving drug inspection by learning from international advanced experiences, such as the Pharmaceutical Inspection Co-operation Scheme (PIC/S), to boost the development of the pharmaceutical industry. In the past, there has been a lack of systematic discussion and comprehensive theoretical construction of the key elements affecting the improvement of China’s Good Manufacturing Practice (GMP) inspection. Through this study, the key elements and theoretical logic for applying PIC/S standards to improve China’s pharmaceutical GMP inspection work are constructed.

**Methods:**

This study uses diverse data and applies grounded theory to construct a model, systematically presenting the key elements and theoretical logic of applying PIC/S standards in improving GMP inspections in China.

**Results:**

The study found that international recognized GMP regulations are external contextual elements, while internal driving elements include professionalization and specialization of inspectors, standardized inspection procedures, and maintain consistent quality levels. The internal dimensions of these four elements are independent, but they can not only individually influence but also partially overlap and have a combined impact Moreover, professionalization and specialization of inspectors, standardized inspection procedures, and maintain consistent quality levels have a dynamic, cyclical promoting effect on the application of PIC/S standards.

**Conclusion:**

Based on China’s national conditions, this study deepens the theoretical framework and content of applying PIC/S standards to improve drug GMP inspections, providing a reference for public sectors to enhance the effectiveness of pharmaceutical regulation.

## 1 Introduction

To meet the needs of the international development of the pharmaceutical industry, the National Medical Products Administration (NMPA) of China is actively applying for membership in PIC/S. As the only international cooperation organization consisting of pharmaceutical regulatory authorities from various countries, PIC/S is dedicated to promoting international exchange and cooperation on GMP standards and unifying inspection standards (PIC/S, 2024; Prasanna Gayathri and Kamaraj, 2024; Brunner, 2004). China’s drug GMP inspection refers to the GMP compliance inspection conducted by the regulatory authorities on the drug marketing authorization applicant (or drug marketing authorization holder) who holds the drug production license and its entrusted production enterprises according to the GMP requirements (National Medical Products Administration, 2021-05; Balzano, 2024). Over the years, NMPA has improved the drug GMP inspection system and management framework by adopting practices from PIC/S and has strengthened the inspector workforce. The application of PIC/S standards will continue to drive China to enhance the existing GMP implementation standards, improve the quality and safety of pharmaceutical production, and promote international pharmaceutical trade and cooperation (Yi and Lin, 2006; Niansu and Yi, 2019; Jun et al., 2024; Ramireddy-Navya et al., 2020). Therefore, conducting research in this field will help China’s pharmaceutical regulatory authorities to plan strategies to enhance drug inspection levels holistically and provide theoretical support for the successful application to join PIC/S. This study analyzes and codes data from multiple sources and uses grounded theory to explore the key elements of applying PIC/S standards to improve China’s GMP inspections. It uncovers the theoretical logic between these key elements and how they influence regulatory decisions. By developing theories within the context of this study, a systematic theoretical framework is provided for improving China’s GMP inspections using PIC/S standards.

This study has both theoretical contributions and practical value. On the one hand, most existing studies adopt a single perspective, focusing on specific factors such as PIC/S technical standards or PIC/S inspector training (Yi and Lin, 2006; Wenhui et al., 2017; Ze et al., 2023; Tang et al., 2023; Sun et al., 2023; Wu et al., 2024), lacking exploration of the relationships between factors. This paper, however, innovates in both research methods and theory. It uses grounded theory to comprehensively summarize and analyze the existing practices of applying PIC/S standards to improve China’s GMP inspections for the first time, helping deepen academic understanding of the characteristics of China’s pharmaceutical inspections. It provides a theoretical framework for understanding the underlying logic of applying PIC/S standards to improve China’s drug GMP inspections and expands the theoretical system of pharmaceutical regulation research. On the other hand, in terms of practical contributions, this study offers strategic recommendations for China’s pharmaceutical regulatory authorities to improve GMP inspections and provides a reference for aligning China’s drug inspection system with international standards.

This paper consists of six sections. Apart from the current section, the other sections are: the second section is a literature review, which organizes and discusses the existing literature in this research field; the third section is research design, which introduces the research methods, sample selection, and data collection; the fourth section is the coding process, which includes open coding, axial coding, selective coding, model construction, and theoretical saturation test; the fifth section is model elaboration, which elaborates on the main and secondary categories of the model, as well as the logical relationships between the main categories and core categories, and among the main categories themselves; the sixth section is research conclusions and future outlook, which presents the conclusions of this study, practical enlightenment, and future outlook. PIC/S is dedicated to promoting international exchange and cooperation on GMP standards and unifying inspection standards.

## 2 Literature review

There are three main research directions concerning the application of PIC/S standards to improve China’s drug GMP inspection work:

First, the focus is on the role and procedure of applying for PIC/S membership. Academics have pointed out that meeting PIC/S requirements is one of the important foundations and prerequisites for aligning pharmaceutical regulation with international standards (Yongxia et al., 2019; Noriko, 2014). PIC/S will play a positive role in promoting NMPA’s participation in international mutual recognition agreements (MRAs), GMP inspection mutual recognition, sharing international experience and technology, and improving the quality of pharmaceutical production (Yi and Lin, 2006; Niansu and Yi, 2019; Scione et al., 2024; Nahler, 2009). The application process for joining PIC/S is divided into two steps: the pre-application and formal application. The globally used evaluation tool “PIC/S Audit Checklist” is used to assess the inspection system of the applying agency.

Second, based on the comparison and summary of the differences between PIC/S GMP and other GMPs, suggestions have been made for regulatory authorities to improve GMP standards and annexes. Scholars from China, South Korea, India and Thailand have compared their countries’ or the World Health Organization’s GMP with PIC/S GMP. They not only acknowledge that PIC/S GMP represents international high-level pharmaceutical production quality management standards, but also believe that their own countries’ GMP should be improved to align with PIC/S GMP (Zhang, 2010; Rushvi patel Charmy-Kothari. Regulatory Need: Harmonized PIC/S GMP and Its Overview and Comparison with WHO GMP, 2015; Ban et al., 2019; Waranon, 2019). Japanese scholar Masahiro Wada pointed out that although the United States and Japan are members of PIC/S, they have different GMP regulations, and the strictness of inspections may also differ. He emphasized the urgent need for the implementation of a unified standardized GMP, given the lack of a dispute resolution mechanism between PIC/S member countries and the non-uniformity of international GMP standards (Wada et al., 2021). Chinese scholars have conducted GMP annex comparison studies in specific areas of drug inspection that are of global concern. They noted that the PIC/S sterile annex has a broader scope of application, emphasizes the use of new tools such as contamination control strategies, provides more detailed and practical clauses, introduces new technologies, and encourages new methods. They also strive to synchronize with existing regulations like ISO 14644 (Tang et al., 2023). They concluded that the advanced therapy products annex in PIC/S has different requirements in terms of scope, principles, quality management, personnel, facilities and equipment, and production management compared to China’s guidelines for cell therapy product manufacturing, and suggested that China should adopt PIC/S concepts and implement inspection scales adapted to product risks (Sun et al., 2023). They did a comprehensive comparison between China’s GMP appendices for Chinese herbal pieces, traditional Chinese medicinal preparations, and relevant regulations with the PIC/S GMP Guide (PE 009-14) Annex 7 on the manufacture of herbal medicinal products and demonstrated that China has established a comprehensive regulatory framework for traditional Chinese medicine (Wu et al., 2024). They also studied the requirements of PIC/S GMP’s Real Time Release Testing (RTRT) annex, namely, Annex 17, and suggested that China should learn and study to introduce a r RTRT guide (Ze et al., 2023).

Third, research on improving the supporting mechanisms for GMP inspections is increasing. China can draw on PIC/S’s experience in optimizing inspection processes (Cheng et al., 2023), enhancing inspector capabilities (Wenhui et al., 2017; Haishan et al., 2024), and improving the quality system construction of inspection agencies (Zhu et al., 2023). Measures such as diversified training methods, training trainers, and establishing advisory inspection programs can be taken to improve the GMP inspector training system (Wenhui et al., 2017). Improvements can also be made in aspects such as formulating risk-based inspection plans, detailing inspection procedures, and ensuring inspection resources, to unify and standardize the operational mechanisms and standards of regulatory agencies for drug inspections (Cheng et al., 2023; Zhu et al., 2023).

Although previous studies on the application of PIC/S standards to improve drug GMP inspection work have provided valuable references for pharmaceutical regulatory practices, there are certain shortcomings. Most related studies compare the practices of China and PIC/S, simply introducing measures that can be adopted by referencing PIC/S experiences. However, they fail to form a systematic theoretical analysis framework based on China’s national conditions, lack the necessary theoretical foundation for support (Tang et al., 2023; Sun et al., 2023; Wu et al., 2024), and have not effectively, systematically, or comprehensively summarized all the key elements in the application of PIC/S standards to improve China’s drug GMP. Moreover, there has been a lack of thought and exploration regarding the logical relationships between these key elements, leading to a continued absence of effective policy proposals (Zhang, 2010; Cheng et al., 2023; Zhu et al., 2023). Given the need for both theoretical and practical research, a systematic theoretical framework for applying PIC/S standards to improve drug GMP inspections needs to be established. This framework should not only cover the key elements but also clarify the relationships and logic between them. Grounded theory, based on the systematic collection of data, can identify core concepts that reflect the essence of phenomena and build new theories through the connections between concepts. This approach is holistic and comprehensive (Glaser And Strauss, 1967), and can compensate for the lack of systematic and theoretical aspects in previous research in this field. Therefore, this study applies grounded theory in line with China’s national conditions, analyzing and coding data from multiple sources to construct a theoretical model of the key elements and logic for applying PIC/S standards to improve China’s drug GMP inspection work, aiming to provide valuable references for improving China’s pharmaceutical inspection system.

## 3 Research design

### 3.1 Research method

**FIGURE 1:**
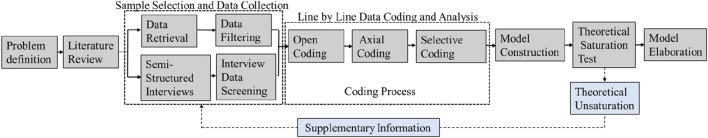
Research flowchart.

Grounded theory is a mature qualitative research method proposed by American scholars Barney Glaser and Anselm Strauss. It emphasizes the use of inductive methods to explore and construct social phenomena in natural settings. This approach effectively explains causal relationships of events and constructs new theories (Chen, 1999). Grounded theory is particularly suitable for descriptive explanations of social phenomena with specific “process” and “interaction” characteristics. Given that the topic of this research not only involves exploring “process-related” issues but also focuses on constructing theories to translate international advanced experiences into practical improvements in China’s drug GMP inspection practices, grounded theory is ideal for examining the complexity of phenomena. It helps uncover how different dimensions are connected, derive actionable measures from past experiences, and enable theory to guide practice. Therefore, this study is well-suited for the use of grounded theory. As shown in Figure 1, the process involves three levels of coding to analyze and summarize the textual material: open coding, which encodes all selected texts sentence by sentence and event by event to identify some themes and categorize them; axial coding, which induces and extracts main categories from the previous stage of coding; selective coding, which organically associates main categories and constructs a theoretical model (Glaser And Strauss, 1967; Corbin and Strauss, 2015).

### 3.2 Sample selection and data collection

The sample selection followed a combination of purposive sampling and convenience sampling principles, collecting two types of data: primary data (interview data) and secondary data.

For primary data, experts familiar with pharmaceutical inspection work were selected as subjects for semi-structured interviews. These experts are primarily five GMP inspectors from drug inspection agencies in region of China with developed biopharmaceutical industries. Due to the professional and specialized nature of drug inspection work, the interviewees were selected based on the criteria that they had at least 5 years of full-time experience in drug GMP inspections and had an understanding of PIC/S working standards, with consistent inspection regulations, inspection targets, and inspection methods. The interviews lasted an average of 40–60 min (Table 1). Following the principle of theoretical saturation, coding was conducted after each interview, and the results were compared with previous interview records. Data collection continued until no new concepts or categories emerged. In total, interviews were conducted with five GMP inspectors (Table 2).

**TABLE 1:** Interview question guide.

Item numbers	Question
1	What do you think are the main differences between PIC/S and China’s current drug GMP inspection standards?
2	What specific improvement measures or strategies do you think can be immediately implemented?
3	What role do you think regulatory agencies should play in applying PIC/S standards to improve drug inspection work?
4	Can you share some successful cases of implementing PIC/S standards in China’s drug GMP inspections?

**TABLE 2:** Basic information of experts in semi-structured interviews.

Item	Category	Number	Composition/%
Expert Category	National Drug GMP Inspectors	3	60%
	Provincial Drug GMP Inspectors	2	40%
Gender	Male	2	40%
	Female	3	60%
Age	35–45 years	4	80%
	45–55 years	1	20%
Education Background	Bachelor	1	20%
	Master	2	40%
	Doctorate or above	2	40%
Professional Background	Pharmacy	3	60%
	Biology	1	20%
	Chemistry	1	20%
Title	Associate Senior	5	100%

For secondary data, literature, news reports, and policy documents were collected through databases such as CNKI (China National Knowledge Infrastructure), Web of Science, and Google Scholar. The search used keywords such as “PIC/S standards”, “drug GMP inspections” and their full names, synonyms, and related terms. To avoid missing relevant articles, sentence searches with the same keywords were also conducted. A total of 59 articles were collected. The screening criteria for the literature were that the paper topics must be related to “PIC/S standards” and “drug GMP inspections,” and any irrelevant papers were excluded. Ultimately, 55 valid articles were retained. News reports primarily came from the top ten influential professional financial media in China in recent years, such as China Business News, covering news related to China’s application to join PIC/S. policy documents were mainly downloaded from chinese government official websites, PIC/S websites, or obtained from pharmaceutical regulatory agencies (Table 3). Additionally, one-third of the original data was randomly selected for theoretical saturation testing.

**TABLE 3:** Data sources.

Data type	Data sources	Data quantity
Literature	https://www.cnki.net/ https://scholar.scholar-xm.top/	55
News reports	“China Business News” “Economic Information Daily” “Health Times”	3
Policy and regulatory documents	National medical products administration official website, PIC/S website	5
Semi-structured interviews	The interview data texts are sourced from national and provincial drug GMP inspectors	5

## 4 Coding process

In accordance with the principles of Grounded Theory, the selected texts were numbered, and the extracted codes were subjected to open, axial, and selective coding. During the coding process, concepts and categories were extracted through expert consultation, repeated thinking, and comparison, core categories were refined, and finally, a model diagram was constructed based on the connection between categories.

### 4.1 Open coding

Open coding is the process of progressively conceptualizing and categorizing qualitative data, forming more concepts and categories through repeated comparisons. First, the interview transcripts were conceptualized sentence by sentence without subjective bias or preset assumptions, and each sentence was coded into free nodes while retaining the original meaning as much as possible (Chen, 1999). Next, nodes that were less relevant to drug GMP inspections were removed, and redundant nodes were merged. For example, “ Conduct pre-inspection risk assessment” was abstracted from the original sentence: “ The PIC/S Risk-Based GMP Inspection Planning Model conducts site risk assessments by inputting the inherent risk information collected during the current inspection and the GMP compliance status of the site from the previous inspection “. This process resulted in 48 initial concepts. Concepts with the same focus were clustered and refined into categories, such as combining the initial concepts of “developing inspection strategies” and “pre-inspection risk assessment” into the category of “develop Risk-based inspection plan,” resulting in a total of 14 categories. Due to space constraints, examples of open coding are shown in Table 4.

**TABLE 4:** Examples of open coding and category extraction.

Category	Initial concepts	Original statements
Compare differences in GMP standards	Analysis of standard differences	Analyzing and studying the differences between the current domestic GMP Annex for Sterile Medicinal Products and the newly revised PIC/S GMP Annex 1 will help inspectors fully understand the new regulatory and technical requirements in the field of sterile medicinal products, and further explore improvements in sterile product inspection practices
	Expansion of standard Application Scope	The scope of application is broader. The China annex applies to sterile preparations and sterile active pharmaceutical ingredients, while the PIC/S Annex for Sterile Products is no longer limited to sterile medicines. It also applies to other non-sterile products, such as liquid products, creams, ointments, and biological products, which are necessary to control and reduce microbial contamination, endotoxins/pyrogens
	⋮	⋮
Dynamically update GMP	Major issues arising from GMP Differences	The case of Leuplin^®^ indicates that, despite the United States and Japan being members of PIC/S, they may have different GMP regulations. Clearly, differing regulations remain a significant issue
	Revising GMP standards	The frequently revised GMP standards by PIC/S can promptly adopt and integrate the latest concepts and management models in the field of pharmaceutical manufacturing
	⋮	⋮
Emphasis on Quality Risk Management (QRM)and Contamination Control Strategies (CCS)	Emphasis on quality risk management	The PIC/S ATMP Annex mentions the QRM principles a total of 33 times, with the implementation of QRM extending deeply into each module
	Emphasis on contamination control strategies	Especially, the PIC/S Aseptic Annex first introduces the concept of CCS, centralizing the scattered contamination control strategies found in our country’s aseptic annex, and proposes targeted provisions
	⋮	⋮
Develop inspection plans based on risk	Formulate inspection strategies	It is possible to refer to PIC/S assessment indicators and guidelines, combine them with the actual situation of China’s drug regulatory system, issue relevant guidelines, establish unified principles, and optimize the mechanism for formulating inspection strategies
	Conduct pre-inspection risk assessments	The PIC/S Risk-Based GMP Inspection Planning Model conducts site risk assessments by inputting the inherent risk information collected during the current inspection and the GMP compliance status of the site from the previous inspection
Standardize inspection reports	Unify the format of inspection reports	PIC/S requires inspection reports to use a unified format. It is recommended that national drug regulatory authorities refer to PIC/S requirements and promptly issue relevant requirements for writing GMP compliance inspection reports, unifying the format and style of GMP inspection reports nationwide
	Develop guidelines for report writing	National and provincial drug regulatory agencies each have their own requirements for report writing, lacking unified guiding documents. There is a shortage of inspection report writing guidelines, and the formats used by various provinces are not consistent
Clarify inspection procedures	Develop inspection procedures	The specific operational procedures are unclear, and the requirements from various provincial bureaus are not unified; furthermore, there are no corresponding standardized operational procedures for administrative measures related to inspection results
	Provide on-site inspection guideline documents	To ensure that all member organizations can conduct inspections according to unified procedures and arrive at consistent GMP compliance conclusions, PIC/S provides an inspection memo for inspectors to reference
Manage inspection data through information technology (IT)	Unify the archival management of inspection information	Establish a dedicated archive to manage and unify the filing of previous GMP inspection information, making it easier to access and query
	Update the inspection classification database	China’s GMP inspection information system is not fully developed, primarily due to an incomplete database. PIC/S requires its members to have procedures for storing inspection data, making it crucial to update the inspection and testing classification database to reflect the current compliance status of enterprises accurately
Communicate risk factors information	Internal communication within regulatory agencies	Developing a GMP inspection plan based on risk requires timely input of risk factors from data across multiple regulatory departments. It is recommended to enhance information sharing and communication between various regulatory departments to effectively assess risks
	Communication between regulatory agencies and enterprises	PIC/S emphasizes that during the development and evaluation phases of real-time release testing, enterprises need to communicate with relevant regulatory authorities. This communication plays a crucial role in the approval and whole lifecycle regulation of the product in the future
	⋮	⋮
Expand the inspector workforce	Strengthen the provision of full-time inspectors	Considering the existing personnel at various levels of drug regulation and staffing allocations, it is important to further strengthen the allocation of full-time national and provincial drug inspectors to adequately meet operational needs
	Supplement with part-time inspectors	Personnel with qualifications for GLP or GMP inspection, managed by market regulatory authorities at all levels, can serve as national and provincial part-time inspectors. Additionally, qualified individuals from related research institutions, inspection and testing organizations, and higher education institutions can be employed as part-time inspectors, providing a vital supplement to the full-time inspector workforce
	⋮	⋮
Systematic training and education	Diversified training methods	PIC/S provides an important training and learning platform for GMP inspectors through various means such as periodic and continuous high-level forums, seminars, expert circles, participation in joint observation projects, mentorship inspection programs, and auditor training
	Training effectiveness evaluation	China lacks an evaluation system for assessing the training effectiveness of inspectors
	⋮	⋮
Reasonable allocation of inspection resources	Reasonable distribution of inspection tasks	Build a professional, diversified, and international inspector data platform that can not only facilitate the reasonable allocation of inspection tasks but also enhance the quality of inspections
	Rational utilization of inspectors	On the other hand, it can dynamically adjust the workload of each inspector, scientifically and rationally utilize the limited number of inspectors, and achieve optimal allocation of resources
Scientific management of inspectors	Hierarchical management of inspectors	It’s necessary to scientifically manage and evaluate the work of inspectors by designing reasonable assessment indicators and implementing corresponding rewards and penalties. Through hierarchical management, inspectors should have opportunities and pathways for grade promotion
	Assess and reward/punish inspectors	Part-time inspectors, not being permanent staff of various drug inspection agencies and having varying capabilities, face challenges in establishing a complete and effective management and assessment system. The inability to implement and quantify rewards and penalties is not conducive to the long-term development of the inspector workforce
Control inspection quality	Evaluate inspection performance	In terms of inspection performance standards and assessments, the PIC/S audit checklist requires inspection agencies to establish GMP inspection performance evaluation indicators (KPIs), such as report issuance times and the ratio of inspection plans implemented/completed, and to conduct annual reviews of these KPIs
	Grasp consistent inspection yardstick	Additionally, there are differences in the understanding and grasp of laws, quality management norms and technical standards, along with issues such as excessive discretionary power. This leads to inconsistencies in the level and quality of inspections among different inspection agencies
Establish a quality management system	Develop quality system documentation	Evaluate whether regulatory agencies have established a quality management system that covers all parts of the GMP compliance program according to international standards, and verify if the quality management system is described in the quality manual and other internal documents of the regulatory department
	Implement quality management	Verify that the quality management system is documented, and that it is followed at both the national and local levels (if applicable), ensuring that consistent quality standards are achieved and maintained

### 4.2 Axial coding

Axial coding aims to further summarize and rearrange the categories derived from open coding by establishing connections between different categories through cluster analysis and developing main categories. The specific approach involves developing the properties and dimensions of the categories to make them more precise, and linking independent categories to discover and establish potential logical relationships between them (Corbin and Strauss, 2015). In this stage, categories are classified based on their logical internal connections at the conceptual level. For example, the four categories—“ expand the inspector workforce”, “systematic training and education”, “reasonable allocation of inspection resources” and “scientific management of inspectors”were summarized into the core category “Professionalization and specialization of inspectors”. This process led to the refinement of four core categories (Table 5).

**TABLE 5:** Refinement of axial coding.

Main category	Category	Connotation explanation
International Recognized GMP Regulations	Compare differences in GMP standards	Analyze the differences of GMP between China and PIC/S, and research to determine the direction for improvements in GMP standards
	Dynamically update GMP	Referencing PIC/S practices, update and adjust GMP standards according to new trends and developments in global pharmaceutical regulation
	Emphasize QRM and CCS	Emphasize the concepts of quality risk management and contamination control strategies in GMP standards and appendices, clarifying the methods of application and implementation focuses of quality risk management
Standardized inspection procedures	Develop risk-based inspection plans	Optimize the mechanism for formulating inspection strategies, scientifically organize the collection of risk factors before inspections, and develop inspection plans based on risk assessment.。
	Standardize inspection reports	Standardize the format and style of GMP inspection reports,promote drug inspectors from various regions implement inspection standards commensurate with product risks. by
	Clarify inspection procedures	Clarify the specific operations of inspections and standardize the administrative procedures for handling inspection results
	Manage inspection data through IT	Centralize the management of inspection information, establish a previous inspection database, and update the categorized inspection database
	Communicate risk factors information	Facilitate information sharing and communication, particularly regarding risk factors, between countries and regions, among various regulatory departments, and between regulatory agencies and enterprises
Professionalization and specialization of inspectors	Expand the inspector workforce	Expand the team of full-time and part-time drug inspectors and promote the professionalization and specialization of inspectors
	systematic training and education	Establish a standardized training system for inspectors, train instructors, adopt diversified training methods such as expert exchanges and seminars, and evaluate training effectiveness
	Reasonable allocation of inspection resources	Establish a Chinese Inspector Information Management Platform to allocate inspection tasks reasonably and achieve nationwide integration and sharing of inspection resources
	Scientific management of inspectors	Implement a hierarchical management system for inspectors, conduct scientific management and evaluation of their work, and establish a comprehensive mechanism for management, assessment, rewards, and penalties
Maintain consistent quality standards	Control inspection quality	Develop performance evaluation indicators for GMP inspections, establish an inspection quality evaluation system, maintain consistent inspection yardstick, and promote uniform inspection levels and quality across national inspection agencies
	Establish a quality management system	Develop quality system documents based on international standards, implement the quality management system, and maintain consistent quality standards

### 4.3 Selective coding and model construction

Selective coding aims to extract the “core category” from the main categories and systematically analyze the relationships between the core category and other categories, thereby developing a theoretical framework. The representative relationship structure of the main categories in this study is listed in Table 6. Through repeated reflection and comparison of the main categories, categories, and interview data, it was found that all the main categories obtained from axial coding represent different characteristics, and without any one of them, the interview data could not be fully covered (Glaser and Strauss, 1967). Ultimately, this study categorized all phenomena under the “Key Elements and Theoretical Logic of Applying the PIC/S Standards to Improve China’s Drug GMP Inspection Work,” positioning it as the core category.

**TABLE 6:** Examples of paradigmatic relationships.

Typical relationship structure	Connotation of relationship structure
International Recognized GMP Regulations — Application of PIC/S Standards to Improve China’s Pharmaceutical GMP Inspections	Compare Differences in GMP Standard, Dynamically Update GMP, and Emphasis QRM and CCS are External Contextual Factors for the Application of PIC/S Standards to Improve China’s Pharmaceutical GMP Inspections
Standardized Inspection Procedures — Application of PIC/S Standards to Improve China’s Pharmaceutical GMP Inspections	Develop Risk-Based Inspection Plans, Standardize Inspection Reports, Clarify Inspection Procedures, Manage Inspection Data through IT and Communicate Risk Factor Information are Essential Internal Operational Processes for Applying PIC/S Standards to Improve China’s Pharmaceutical GMP Inspections
Professionalization and Specialization of Inspectors — Application of PIC/S Standards to Improve China’s Pharmaceutical GMP Inspections	Expand the Inspector Workforce, Systematic Training and Education, Reasonable Allocation of Inspection Resources, and Scientific Management of Inspectors are the Internal Intellectual Support and Talent Assurance for Applying PIC/S Standards to Improve China’s Pharmaceutical GMP Inspections
Maintaining Consistent Quality Standards — Application of PIC/S Standards to Improve China’s Pharmaceutical GMP Inspections	Establish a Quality Management System and Control Inspection Quality are Internal Control Factors and Desired Outcomes for Applying PIC/S Standards to Improve China’s Pharmaceutical GMP Inspections
Standardized Inspection Procedures — Professionalization and Specialization of Inspectors — Application of PIC/S Standards to Improve China’s Pharmaceutical GMP Inspections	Standardized Inspection Procedures Are Executed by Professionalized and Specialized Inspectors to Achieve the Application of PIC/S Standards in Improving China’s Pharmaceutical GMP Inspections
Maintaining Consistent Quality Standards — Standardized Inspection Procedures — Application of PIC/S Standards to Improve China’s Pharmaceutical GMP Inspections	Maintaining Consistent Quality Standards Requires the Development of Comprehensive Standardized Inspection Procedures to Achieve the Application of PIC/S Standards in Improving China’s Pharmaceutical GMP Inspections
International Recognized GMP Regulations — Professionalization and Specialization of Inspectors — Application of PIC/S Standards to Improve China’s Pharmaceutical GMP Inspections	International Recognized GMP Regulations Are Implemented by Professionalized and Specialized Inspectors Who Strictly Adhere to Legal Norms During Inspections to Achieve the Application of PIC/S Standards in Improving China’s Pharmaceutical GMP Inspections

**FIGURE 2:**
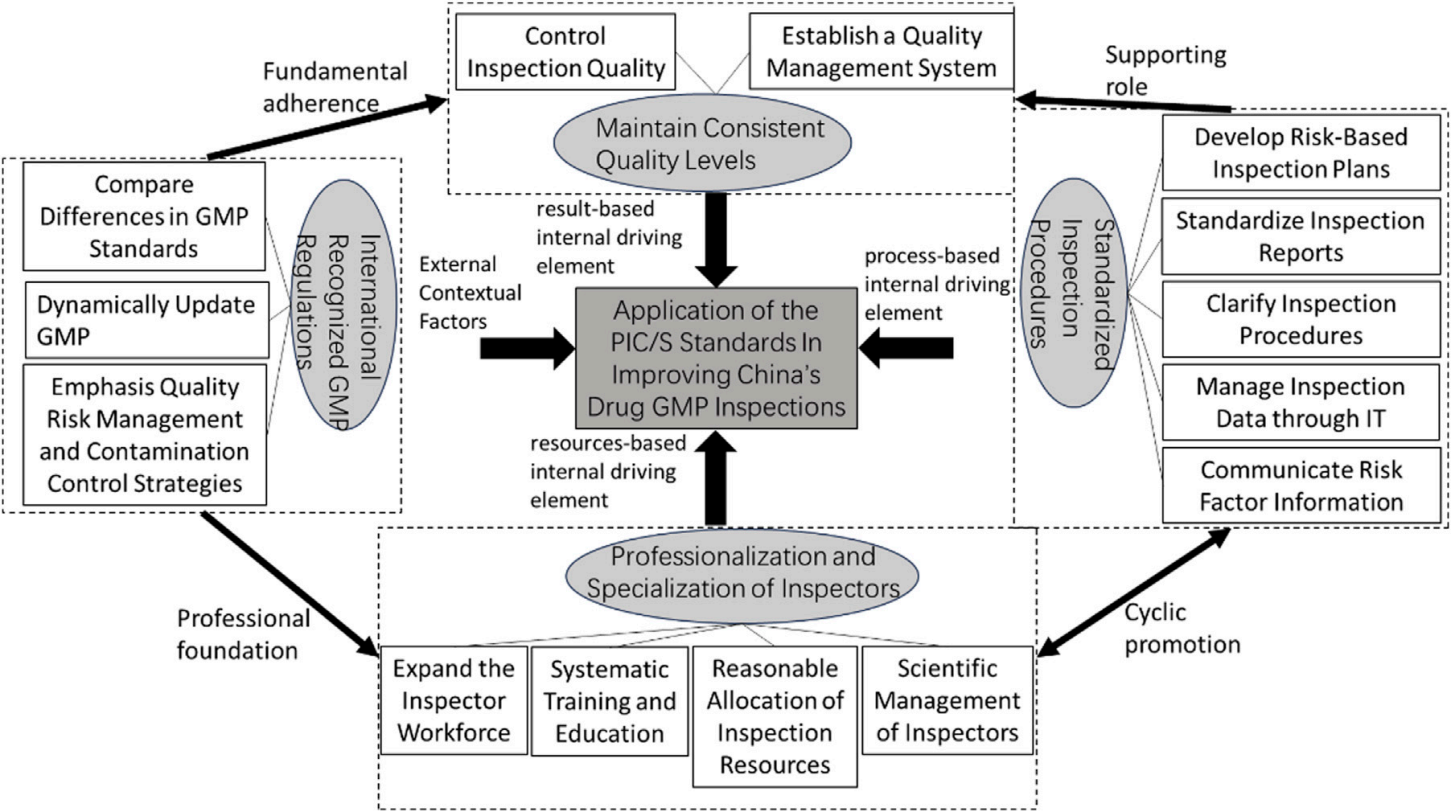
The key elements and theoretical logic model of applying PIC/S standards to improve China's drug GMP inspection.

Around the core category, its “storyline” structure is as follows: International recognized GMP regulations serve as the external contextual factor for the application of the PIC/S standards in improving China's drug GMP inspection work, driving further international coordination of China's drug production inspection laws and regulations to improve drug inspection work. The professionalization and Specialization of inspectors, standardized inspection procedures, and maintain consistent quality levels are resources-based, process-based, and result-based internal driving elements respectively. The professionalization and specialization of inspectors provides continuous human and intellectual support for drug GMP inspections; standardized inspection procedures enhance the normativity and scientificity of GMP inspections; maintain consistent quality levels ensures quality control throughout the entire process and in the final results. This leads to the construction of the key elements and theoretical logic model for applying the PIC/S standards to improve China's drug GMP inspection (See Figure 2 for details).

### 4.4 Theoretical saturation test

To ensure the scientific rigor of the grounded theory research process and the accuracy of the research results, one-third of the original data was randomly selected for theoretical saturation test. The same steps—open coding, axial coding, and selective coding—were performed again. After analysis, no new concepts or categories were found, and no new relationships between the categories were identified. Additionally, the coding results and the model were reviewed by three experts in the field, and their positive feedback confirmed that the theoretical model passed the theoretical saturation test.

## 5 Model elaboration

Through the above coding and analysis, a key elements and theoretical logic model for the application of PIC/S standards to improve China’s pharmaceutical GMP inspection work has been established. The four key elements are independent within each dimension; they can not only influence individually but also partially overlap to jointly impact the application of PIC/S standards in improving China’s pharmaceutical GMP inspection. The key to maintaining consistent quality levels depends on whether the GMP regulations are effectively executed by the inspectors. Therefore, international recognized GMP regulations serve as the professional foundation for inspector professionalization and specialization, and the fundamental guide for maintaining consistent quality levels. The overlapping of inspector professionalization and specialization with maintaining consistent quality levels produces a combined effect. The implementation of standardized inspection procedures at each stage requires continuous enhancement of the inspectors’ professional and technical capabilities. In this study, inspector professionalization and specialization accompany the entire process of applying PIC/S standards to improve China’s GMP inspections and influence the GMP inspection cycle through standardized inspection procedures. Therefore, standardized inspection procedures and inspector professionalization and specialization play a dynamic, cyclical, and mutually reinforcing role in improving GMP inspections. They safeguard the maintenance of consistent quality levels and produce a combined effect when overlapped with the goal of maintaining consistent quality levels.

### 5.1 International recognized GMP regulations

Internationally Recognized GMP regulations are external contextual factors for the application of PIC/S standards to improve China’s pharmaceutical GMP inspections. These include compare differences in GMP standards, dynamically update GMP, and emphasis on QSM and CCS.

First, PIC/S considers internationally coordinated GMP as an important indicator of regulatory authority for pharmaceutical regulatory agencies (Tianfang et al., 2022). Comparing GMP standard differences is an international practice (Zhang, 2010; Ban et al., 2019) and is also the most crucial method for China to apply PIC/S standards in improving GMP inspections. By exploring the equivalence between GMP and PIC/S GMP technical requirements, China can identify gaps between its own technical standards and internationally advanced GMP, laying a foundation for revising and improving GMP and its appendices, thus advancing the alignment of GMP inspections with international standards. Singapore and Malaysia were once cited by PIC/S for their regulatory requirements differing from those of PIC/S (Hu and Wu, 2023), which shows that PIC/S uses the comparison of GMP standard differences as one of the criteria for determining whether a regulatory agency is authoritative. In 2023, the Center For Food and Drug Inspection of NMPA (CFDI) led a study comparing GMP technical requirements with those of PIC/S to provide technical support for enhancing the current GMP level.

Second, dynamic updates to GMP involve adjusting GMP regulations in response to new global pharmaceutical regulatory trends and challenges, helping China identify regulatory challenges in a timely manner and adjust its standards flexibly. This, in turn, enhances the timeliness and sustainability of applying PIC/S standards to improve GMP inspections. China’s current GMP version is the 2010 edition, which has undergone only two revisions (Pharmaceutical Inspection Co), while the latest PIC/S GMP guidelines, effective from August 2023, are the PE009-17 version, having undergone 18 revisions (Zhang and Dong, 2023). Therefore, China, by formally applying to join PIC/S, aims to further improve the pharmaceutical GMP standard update mechanism through the completion of a gap analysis report on GMP and appendices with PIC/S and the formulation of revision plans.

Third, the emphasis on QRM and CCS focuses on the application of the Quality Risk Management principle and Contamination Control Strategy. The new PIC/S GMP Appendix 1 not only places greater emphasis on QRM but also mentions CCS 51 times throughout the text, incorporating CCS requirements throughout the pharmaceutical production process (Wang and Yang, 2023). This international standardization has drawn significant attention from global pharmaceutical companies and has provided clear direction for China’s efforts to improve pharmaceutical GMP inspections. Pharmaceutical inspection agencies and industry players have already started related research (Yan, 2023; Xiang and Wang, 2024). Recently, the China Pharmaceutical Equipment Engineering Association led the formulation of the group standard “Technical Guide for Contamination Control Strategies (CCS) in aseptic drug Production” (T/CPAPE 01–2024), which officially came into effect on 1 June 2024, indicating China’s keen interest in learning from and applying PIC/S international standards. This sets the foundation for the future update of GMP concepts and further improvement of pharmaceutical inspection practices (Tang et al., 2023).

In summary, comparing standard differences is a prerequisite for achieving international coordination of GMP regulations, dynamic GMP updates is a mean to achieve this, and the emphasis on QRM and CCS is the latest strategy to implement it. Internationally recognized GMP regulations provide a standardized and coordinated international framework for pharmaceutical GMP inspections, making them the most important measure for China in applying PIC/S standards to improve GMP inspections.

### 5.2 Standardized inspection procedures

Standardized inspection procedures, which integrate develop risk-based inspection plans, standardized inspection reports, clarify inspection procedures, manage inspection data through IT, communicate risk factor information are key internal contextual factors for applying PIC/S standards to improve China’s pharmaceutical GMP inspections.

First, develop risk-based inspection plans involves assessing risk factors before the inspection to reasonably determine the scope, focus, frequency, and depth of the inspection. This risk management-based mechanism helps improve the efficiency of identifying risks and problems during inspections, while also saving inspection resources. It is crucial for enhancing the targeting of GMP inspections in China. In 2021, China introduced the “Pharmaceutical Inspection Management Measures (Trial)” (National Medical Products Administration, 2021-05), requiring drug regulatory departments to develop inspection plans based on risk principles. In 2023, this normative document was revised to align more closely with the requirements of PIC/S and other international organizations (National Medical Products Administration, 2023-07), including the use of specific risk assessment factors to define inspection items, timing, and methods. This revision offers concrete guidance for applying PIC/S standards in risk-based GMP inspections.

Second, standardize inspection reports involves unifying the format and writing requirements for inspection reports. At present, pharmaceutical regulatory agencies in China have their own guidelines for writing inspection reports and lack a unified document guide. In contrast, PIC/S requires its members to define and evaluate inspection report formats and contents. Establishing a unified set of guidelines for report writing across China will improve the key details and consistency of GMP inspections, in line with PIC/S standards.

Third, clarify inspection procedures means establishing clear operational steps for inspections and standardized procedures for dealing with inspection outcomes. Scientific and robust regulations are essential for effective risk identification. Scientific and sound regulations and systems are a necessary prerequisite for risk identification because only by standardizing the scope and standards of inspections nationwide and formulating unified risk judgment principles and guidance can a consistent “yardstick” be provided for risk identification. PIC/S requires member agencies to define detailed inspection procedures, which inspectors must follow. Therefore, defining clear inspection procedures is a critical component for improving China’s GMP inspection work under PIC/S standards. In November 2023, NMPA issued the “Drug GMP Compliance Inspection Work Procedures (Trial)”, further providing unified procedures and requirements for standardizing GMP inspections based on PIC/S concepts.

Fourth, manage inspection data through IT involves the unified filing and management of inspection information, as well as the establishment and timely updating of a categorized database of past inspections, is a key measure for scientifically judging the potential risks in the production process of pharmaceutical companies and reasonably determining the scope and frequency of inspections through risk assessment. PIC/S requires its members to have procedures for storing inspection data. It has also developed a non-profit International Medicinal Inspectorate Database (IMID), which is used to search for GMP compliance status at drug production sites, helping to reduce inspection frequency and eliminate redundant reports (Ban et al., 2019). Promoting data-sharing will significantly improve the efficiency of GMP inspections in the digital age. In 2023, the NMPA issued “Guiding Opinions on Accelerating Provincial Drug Wisdom Supervision”, and opened accounts for provincial drug regulatory bureaus to access the NMPA National Pharmaceutical Smart Regulation Platform.

Fifth, communicate risk factor information refers to the smooth sharing of risk-related information between different national and regional authorities, regulatory departments, and between regulators and enterprises. This is an important prerequisite for pharmaceutical inspection agencies to make risk-based inspection decisions and identify potential risks. Effective risk communication enhances the scientific approach to GMP inspections. The WHO’s “International Health Regulations” identify risk communication as one of the eight core competencies for member countries to manage international health threats (World Health Organization, 2008). China’s territorial management of drug GMP inspections has drawn on the risk communication concepts of PIC/S member countries, continuously improving the mechanisms for analyzing the drug safety situation and consulting on risk between national, provincial regulatory authorities, and local provincial governments (Zhu et al., 2023), and continuously strengthening the horizontal and vertical communication of risk factor information.

In summary, develop risk-based inspection plans is the starting point of the standardized inspection process, clarify inspection procedures is the main content, standardize inspection reports serves as the presentation of the results, manage inspection data through IT is the application requirement of inspection data, and communicate risk factor information runs throughout the process. All of these are essential elements of the standardized inspection procedures. As a key measure for China to learn from and apply PIC/S standards, standardized inspection procedures provide operational norms for inspectors to efficiently carry out pharmaceutical GMP inspections.

### 5.3 Professionalization and specialization of inspectors

The professionalization and specialization of inspectors provide the intellectual support and human resources necessary for improving China’s pharmaceutical GMP inspections through the application of PIC/S standards. This involves expand the inspector workforce, systematic training and education, reasonable allocation inspection resources, and scientific management of inspectors. Given that the implementation of PIC/S standards in GMP inspections requires continuous intellectual support, the professionalization and specialization of inspectors is a critical internal driving force.

First, expand the inspector workforce refers to increasing the number of inspectors to match the workload, which is an important work requirement of PIC/S and provides professional human resource guarantees for China to apply PIC/S standards to improve drug inspections. In 2019, China issued the “Opinions on Establishing a Professionalized and Specialized Drug Inspection Team” (General Office of the State Council, 2019-07), with NMPA taking the lead and various provinces and cities successively issuing related implementation opinions. Over the years, China has built up a professional and specialized team of drug inspectors with full-time inspectors as the main body, supplemented by part-time inspectors, including more than 1,000 national drug GMP inspectors and nearly 7,000 provincial drug GMP inspectors.

Second, systematic training and education means establishing a standardized drug inspector training system, training trainers, adopting diversified training methods such as expert exchanges and discussions, and evaluating training effects to ensure that inspector resources are adapted to the needs of drug inspection work (Yun et al., 2023). This is one of the most important work contents of PIC/S and is increasingly valued by drug regulatory authorities in China (Wenhui et al., 2017), establishing a standardized knowledge update guarantee system for applying PIC/S standards to improve drug inspections. In September 2021, NMPA issued the “Professional and Specialized Drug Inspector Education and Training Management Measures”, focusing on establishing a unified and standardized professional and Specialized drug inspector training system, constructing an integrated education, learning, practice, and inspection training mechanism, promoting the construction of inspector training institutions and training teachers, implementing classified and pre-service and daily training systems for inspectors, and accelerating the establishment of national and provincial drug inspector training bases, establishing joint training mechanisms with colleges, scientific research institutions, relevant international organizations, and non-profit organizations.

Third, reasonable allocation of inspection resources refers to developing a mechanism that matches inspection workload with the available number of inspectors, ensuring that inspection resources are efficiently distributed. This addresses the challenge of limited inspection resources amidst the rapid growth in pharmaceutical product numbers and variety. In 2022, NMPA issued the “Professional and Specialized Drug Inspector Dispatch and Use Management Measures”, forming a pattern of NMPA responsible for the overall management of the country, provincial bureaus responsible for managing within their jurisdictions, and national and provincial drug inspection institutions responsible for their duties. At the same time, CFDI is building a national unified inspector database and inspector information platform to achieve information sharing and coordinated linkage of national and provincial inspectors, striving to overcome the problem of lacking an inspector selection procedure that the U.S. FDA faced when applying to join PIC/S (Hu and Wu); in 2024, NMPA also clarified that provincial regulatory authorities should unify the inspection frequency of inspectors according to PIC/S’s requirements on the matching of the number of inspectors and inspection tasks, requiring at least two drug production inspections per person per year.

Fourth, scientific management of inspectors means classifying and managing inspectors and improving management assessment, reward, and punishment mechanisms, clarifying human resource management principles for applying PIC/S standards to improve drug GMP inspections. To solve the difficulty of assessing and managing inspectors, NAMP has clearly required provinces to set reasonable assessment indicators, establish an assessment and evaluation system based on job responsibilities, focused on performance contributions, combining daily assessments with annual evaluations, and ability evaluations with performance assessments (Di et al., 2023). In 2021, NMPA issued the “Professional and Specialized Drug Inspector Grading and Classification Management Measures”, basically establishing a management system for professional and professional drug inspection teams. In summary, expand the inspector workforc is the first task of the professionalization of Chinese inspectors, systematic training and education is an important mean to achieve the specialization of inspectors, reasonable allocation inspection resources is an effective way to overcome the difficulty of matching the number of inspectors with inspection tasks, and scientific management of inspectors is the main measure of the professionalization of inspectors. The professionalization and Specialization of inspectors is the most important measure for China to draw on PIC/S’s advanced experience to provide intellectual drive and talent support for drug GMP inspections.

### 5.4 Maintain consistent quality levels

Maintain consistent quality levels is both an internal contextual factor and a desired outcome for improving pharmaceutical GMP inspections in China. This includes establish a quality management system and control inspection quality.

First, according to China’s “Pharmaceutical Production Supervision and Administration Regulations”, pharmaceutical inspection agencies are required to establish a quality management system to improve the efficiency and quality of pharmaceutical regulation (State Administration For Market Supervision, 2019-07). The establishment of this quality management system clarifies the quality standards and requirements for applying PIC/S standards to improve pharmaceutical GMP inspections. While PIC/S allows for different management models, such as the “centralized + five regional offices” vertical management approach of the U.S. FDA (Guang, 2016) or Germany’s decentralized model through the Zentralstelle der Länder für Gesundheitsschutz (ZLG) (Jing and Jun 2022), it requires that all pharmaceutical inspection agencies, regardless of region, adhere to international standards and establish a highly unified quality management system. To meet PIC/S evaluation criteria, the NMPA issued the “Provincial Pharmaceutical Inspection Agency Quality Management System Rating Table” in 2024, which includes 11 primary indicators and 84 secondary indicators to accelerate the development of a quality management system in provincial inspection agencies.

Second, control inspection quality refers to establishing a performance evaluation system for GMP inspections through the development of key performance indicators (KPIs) for inspection quality. This system ensures the uniformity of inspection standards across regions, acting as a safeguard for improving GMP inspections through the application of PIC/S standards. Poor inspection quality could allow products with potential risks to enter the market, posing harm to patients. Therefore, maintaining stable inspection quality is crucial (Jing and Jun 2022). PIC/S audits include requirements for reviewing inspection results, such as internal reviews of defects and conclusions, and providing key performance indicators for GMP compliance monitoring (PIC/S). By establishing an evaluation system for GMP inspection quality, this allows for better control of inspection quality and strengthens the central role of GMP inspections in pharmaceutical regulation (Hu and Wu, 2023; Zhang and Dong, 2023).

In conclusion, establish a quality management system that aligns with international standards and is unified nationwide is key to maintain consistent quality levels in China. Control inspection quality is the core measure to ensure this consistency. Maintain consistent quality levels is an intrinsic requirement for promoting mutual recognition of pharmaceutical GMP inspections within China and internationally through the adoption of PIC/S standards.

## 6 Conclusion and Prospects

### 6.1 Conclusions

This study uses grounded theory to code and systematically analyze the textual materials related to the application of PIC/S standards to improve China’s pharmaceutical GMP inspections, and concludes the following:

Based on grounded theory, a model of the key elements and theoretical logic of PIC/S standards applied to improve China’s pharmaceutical GMP inspections was constructed: International recognized GMP regulations are the external contextual elements for the application of PIC/S standards to improve China’s pharmaceutical GMP inspections, promoting the further international coordination of Chinese drug production inspection laws and regulations to improve drug inspection work. The professionalization and specialization of inspectors, standardized inspection procedures, and maintaining consistent quality levels are resource-based, process-based, and result-based internal driving elements, respectively. The professionalization and specialization of inspectors continues to provide human and intellectual support for drug production inspections; standardized inspection procedures enhance the standardization and scientific nature of GMP inspections; maintaining consistent quality levels requires the quality of inspection work to be controllable from the whole process and results. The four elements are internally independent and can not only influence individually but also have a combined impact on the application of PIC/S standards to improve China’s GMP inspections. The professionalization and specialization of inspectors and standardized inspection procedures have a dynamic cyclical promotional effect on the application of PIC/S standards to improve GMP inspections. Most existing studies have adopted a single perspective to focus on PIC/S technical specifications, inspector training, and other individual influencing factors (Balzano, 2024; Wenhui et al., 2017; Ze et al., 2023), and lack a systematic sorting of the relationships between factors (Yongxia et al., 2019; Cheng et al., 2023; Tianfang et al., 2022). However, this study, for the first time, summarizes and analyzes existing practices from an overall perspective, abstracts a model, and provides a more comprehensive theoretical analysis framework for the application of PIC/S standards to improve China’s GMP drug inspections, helping to answer a series of practical regulatory issues.

### 6.2 Practical enlightenment

(1) Benchmark PIC/S to revise GMP, establish a dynamic update mechanism, and upgrade service industry measures. China should accelerate the alignment with PIC/S requirements to modify drug GMP and improve the dynamic update mechanism for standards. After the new GMP is released, to better integrate GMP concepts throughout the entire drug production process, NAMP’s interpretative work can refer to the practice of EMA, which assists enterprises in accurately understanding PIC/S’s GMP by releasing GMP Q&As on its official website, helping the reshaping of the GMP and inspection system in the pharmaceutical industry.
(2) Strengthen the management of professionalized and specialized professional drug inspector teams, and systematically train to improve professional capabilities. According to the theory of learning organizations, drawing on PIC/S’s 8 types of training methods, accelerate the construction of a unified national inspector training system, and strengthen the systematic and continuous training of inspectors throughout their entire career cycle. The national inspection agency should train the inspection teachers of various provinces to ensure the basic unity of depth and frequency of training for inspectors at all levels nationwide (Wenhui et al., 2017). Organize inspectors to actively participate in PIC/S training to align their understanding and grasp of GMP with international levels.
(3) Strengthen information system construction and build an efficient and intelligent information-based drug regulatory system. In modern regulation, massive data is generated at all times, and scientific data is the basis for decision-making. Accelerating the construction of informationization is an urgent need to support China’s drug inspection work. NMPA should lead drug inspection agencies at all levels to accelerate the construction level of informationization to align with PIC/S, establish a unified and efficient national inspection information management platform and inspector management information platform, unify the format of national inspection information releases, improve information sharing and update mechanisms, and increase the completeness of information, advanced search functions, and database usage guides (Niansu and Shunhong, 2019). Identifying risk factors through data and promoting the organic combination of scientific decision-making and the practice of key elements.
(4) Strengthen cooperation and exchanges to promote the mutual recognition of inspection results. Drug GMP inspections involve complex economic interests. According to the stakeholders theory (Wooddj, 1991), it is necessary to strengthen international exchanges, establish good working relationships, strive to join PIC/S as soon as possible, and enable regulatory agencies to handle common problems under the same discourse system, using common rules, standards, and frameworks, providing effective coordination measures for the distribution of interests and sharing of risks in China’s drug participation in international market competition.
(5) Break the drawbacks of the local management model and build a unified quality management system. To maintain the stable operation of drug regulatory agencies and the power to promote the implementation of various works under the concept of “unification,” it is necessary to resolve the issues of differences in inspection standards and non-unified quality management systems across provinces, ensuring the high efficiency, authority, and unity of regulatory agencies, which is beneficial for NMPA to obtain international recognition.

### 6.3 Research prospects

The data for this study come from textual materials related to the application of PIC/S standards to improve China’s pharmaceutical GMP inspections, including literature, policies, news, and expert interviews. Although all possible considerations were made to ensure the comprehensiveness and completeness of the materials during the coding process, and the principle of theoretical saturation was followed, the publication of information itself may still have a certain degree of emphasis and limitations. In the future, methods such as the Delphi method can be used to further verify and supplement the application of PIC/S standards to improve China’s pharmaceutical GMP inspections. At the same time, although this paper discusses the improvement of China’s pharmaceutical GMP inspections on the basis of practical summaries from a theoretical perspective, there is still a lack of related theoretical research. Drug trade internationalization is in line with the general trend of today’s era development. To better serve Chinese drugs going global, more corresponding theoretical guidance is still needed.

## Data Availability

The original contributions presented in the study are included in the article/supplementary material, further inquiries can be directed to the corresponding author.
